# Typing of *Mycobacterium avium* Subspecies *paratuberculosis* Isolates from Newfoundland Using Fragment Analysis

**DOI:** 10.1371/journal.pone.0126071

**Published:** 2015-04-30

**Authors:** Milka P. Podder, Susan E. Banfield, Greg P. Keefe, Hugh G. Whitney, Kapil Tahlan

**Affiliations:** 1 Department of Biology, Memorial University of Newfoundland, St. John’s, Newfoundland and Labrador, Canada; 2 Department of Health Management, Atlantic Veterinary College, University of Prince Edward Island, Charlottetown, Prince Edward Island, Canada; 3 Animal Health Division, Newfoundland and Labrador Department of Natural Resources, St. John's, Newfoundland and Labrador, Canada; St. Petersburg Pasteur Institute, RUSSIAN FEDERATION

## Abstract

Short Sequence Repeat (SSR) typing of *Mycobacterium avium* subspecies *paratuberculosis (Map)* isolates is one of the most commonly used method for genotyping this pathogen. Currently used techniques have challenges in analyzing mononucleotide repeats >15 bp, which include some of the *Map* SSRs. Fragment analysis is a relatively simple technique, which can accurately measure the size of DNA fragments and can be used to calculate the repeat length of the target SSR loci. In the present study, fragment analysis was used to analyze 4 *Map* SSR loci known to provide sufficient discriminatory power to determine the relationship between *Map* isolates. Eighty-five *Map* isolates from 18 animals from the island of Newfoundland were successfully genotyped using fragment analysis. To the best of our knowledge, this is the first report on *Map* SSR diversity from Newfoundland dairy farms. Previously unreported *Map* SSR-types or combinations were also identified during the course of the described work. In addition, multiple *Map* SSR-types were isolated from a single animal in many cases, which is not a common finding.

## Introduction

*Mycobacterium avium* subspecies *paratuberculosis* (*Map*) is a slow growing bacterium and is the cause of Johne’s disease, which is associated with chronic debilitating granulomatous enteritis that affects the small intestine of cattle, sheep, goats, farmed deer and {Li, 2005 #1017}other ruminants [1–6]. Johne’s disease is a major cause of concern to the dairy industry and there is also some concern regarding the association of *Map* with Crohn’s disease in humans [7–9]. Treatment of dairy animals infected with *Map* is impractical because it can be only achieved by using a combination of antibiotics, many of which are very expensive, not licensed for food animals and require long term dosing [10]. Therefore, infected animals are culled, which is also a part of the Johne’s disease control/management practice [5]. Because diagnosis is very challenging early on in disease progression, animals can still get infected by *Map* through exposure to other shedding asymptomatic animals and environmental contamination [11,12]. The long incubation period of *Map* and the non-specific clinical symptoms exhibited by infected animals makes the diagnosis, management and control of Johne’s disease difficult. To decrease the spread of Johne’s disease, surveillance programs are being established throughout the world for determining the sources of infections, the prevalence of the causative agent and the relationship(s) between *Map* isolates from dairy farms. Studies are also being conducted to examine the role of host genetics in determining the susceptibility of individual animals and their clinical course once infected. Such programs are vital for devising effective control strategies against this devastating disease [13].

More recently, there have been a number of reports on the molecular epidemiology of *Map*. In previous studies, single or combined molecular methods have been used to obtain epidemiological data regarding *Map* strain types [14–17]. Most of the previously used strain typing methods are expensive, time consuming, lack discriminatory capabilities and sometimes do not provide consistent results [14,15,18]. Despite these limitations, the information obtained from such studies is essential for identifying sources and transmission routes more accurately. When combined with information on host genetics, strain typing studies can also be used to determine strain pathogenicity and host resistance. Molecular techniques, especially DNA short-sequence-repeat (SSR) analysis has been shown to be a powerful tool for discriminating between *Map* isolates at the genetic level [5,14,15,16,18,19]. Due to differences in the numbers of nucleotide repeats associated with SSRs from different *Map* isolates, the relatedness and prevalence of *Map* strains can be monitored within/between farms and the environment [14,19]. One major problem with conventional methods for SSR analyses such as the use of Sanger sequencing, is that they are prone to artifacts and failure due to challenges associated with determining the DNA sequences of the repeats, with the most recent technology being capable of analyzing repeats up to 15 bp using a mass spectrometry based approach [18]. Therefore, there is need for developing cheap, reliable and reproducible methods for *Map* SSR analysis, which can accurately measure repeats over 15 bp in length. Recently, DNA fragment analysis was used for *Map* SSR typing [16]. PCR fragments containing the SSRs were obtained using fluorescently labeled primers and were subjected to capillary electrophoresis for determining their sizes [16]. The island of Newfoundland which situated at the eastern edge of North America has a number of dairy farms. There is significant movement of animals within the Newfoundland dairy industry, with new animals being brought onto the island for entry into the production chain. In addition, some heifers are also shipped to other Atlantic Canada provinces on the mainland for raring, before they return as adult producers, as it is economically more feasible to do so in some situations. Therefore, there is interest in analyzing the diversity *Map* isolates infecting animals from the island for comparison to those found elsewhere in North America. In the current study we used fragment analysis to analyze *Map* isolates from five Newfoundland dairy farms, the results of which are described below.

## Materials and Methods

### Ethics statement

The described study was carried out under a formal agreement between the Dairy Farmers of Newfoundland and Labrador (NL) and the Chief Veterinary Officer for the Province of NL (HGW). The study was approved by the Institutional Animal Care Committee (IACC, Memorial University of Newfoundland) as an “A” rated protocol because the samples used in the study were obtained from routine veterinary diagnostic submissions unrelated to this research. The report describes laboratory microbiological analysis and did not directly involve any animals.

### Media, reagents and culture conditions

All reagents and media used in the study were purchased from Sigma Aldrich, Fisher Scientific or VWR International, Canada, unless otherwise mentioned. DNA oligonucleotide primers were purchased from Integrated DNA Technologies (USA). *Map* cultures were grown at 37°C. Fecal samples from 18 animals displaying varying clinical symptoms of Johne’s disease or suspected of being infected (S1 Table), were collected by the Animal Health Division, Department of Natural Resources, Government of NL and were sent to the Atlantic Veterinary College, University of Prince Edward Island (UPEI) for diagnosis. Trek-ESP II liquid culture using Trek ESP Para-JEM media (Thermo Scientific) was used to culture *Map* from bovine fecal samples as described previously [20], which were verified by acid-fast staining. To confirm the presence of *Map* in the cultures, chromosomal DNA was isolated using the Tetracore *Map* extraction system and was used as template along with the Tetracore VetAlert Johne’s Real-Time PCR kit as per the manufacturer’s instructions (Tetracore, USA). After the described analysis, the culture samples were stored as frozen glycerol stocks and were sent to the Memorial University of Newfoundland for further analysis.

The culture samples from UPEI were streaked out on to Middlebrook 7H11 agar plates supplemented with oleic acid-albumin-dextrose-catalase (OADC) and mycobactin J (2 mg/L, Allied Monitor, USA) to obtain isolated *Map* colonies as described previously [14]. The PANTA (polymyxin B, amphotericin B, nalidixic acid, trimethoprim and azlocillin) antibiotic mixture was also added to the medium to prevent the growth of other contaminating microorganisms [21]. The plates were incubated for 4–6 months until minute colonies were observed, which were confirmed to be *Map* by acid-fast staining. Three to five isolated colonies from each plate (corresponding to each animal) were then used to inoculate separate 5 mL Middlebrook 7H9 broth cultures supplemented with albumin-dextrose-catalase (ADC) and mycobactin J (2 mg/L). To avoid the clumping of cells, culture tubes contained sterile glass beads and were incubated with agitation. Growth was observed for 85 isolates (3–5 isolates sampled from each animal) after 2–3 months of incubation based on an increase in the turbidity of the cultures, which were then used to prepare glycerol stocks for storage and for chromosomal DNA isolation as described below. Acid-fast staining was performed at different stages to ensure that the cultures were axenic.

### Chromosomal DNA isolation, SSR sequencing and fragment analysis

The QIAamp DNA Mini kit (Qiagen) was used for isolating chromosomal DNA from the remaining 3.5 ml 7H9 cultures from above using 0.1 mm zirconia silica beads and a SpeedMill PLUS homogenizer (Analytik Jena, Germany) according to the manufacturer’s recommendations. All PCR reactions were performed using the Phusion High-Fidelity PCR Kit along with 3% DMSO and the GC buffer (New England Biolabs, Inc. Canada). PCR products were visualized by agarose gel electrophoresis, purified using the EZ-10 Spin Column PCR Products Purification Kit (Bio Basic, Canada) and were sent for DNA sequencing or fragment analysis to the Centre for Applied Genomics (TCAG), University of Toronto, Canada. The four SSR loci (L1-L4) previously shown to provide good discriminatory power for subtyping *Map* isolates were chosen for analysis [5,14]. The DNA sequences of the four SSR repeats were determined for a handful of *Map* isolates obtained as part of a separate study as described previously [5,14]. The sequences of the 4 loci for the *Map* K10 strain (genome sequenced) were obtained from previous publications [14,22] and were confirmed using fragment analysis as described below. This was done to determine the exact numbers of the SSR repeats for each of the strains, which were later used as standards during fragment analysis.

For fragment analysis, four primer pairs were designed which were specific for each locus, and one primer from each pair was labeled with 6-fluorescein amidite (6-FAM) to give PCR products ranging from 127 to 255 bp. Primers that have the 6-FAM dye next to a guanine base near the 5' end can have decreased fluorescence. Therefore, either the forward or the reverse primer from each pair was labeled to avoid any complications. The DNA sequences of the primers used for obtaining PCR products for fragment analysis for each locus were as follows: L1 (F: GGTGTTCGGCAAAGTCGTT/R: TTGACGATCACCAGCCCG), L2 (F: TCGCCTCAGGCTTTACTGAT/R: CACGTAGGTCCGCTGATGA), L3 (AGGCCTTCTACGTGCACAAC/R: GAGATGTCCAGCCCTGTCTC) and L4 (F: CTCGTGGAAACCCTCGAC/R: GGTGCTGAAATCCGGTGT). Unpurified PCR products were sent to the TCAG facilities for fragment analysis using the ABI 3730XL or 3100 capillary electrophoresis instruments using the GeneScan 500 ROX Size Standard, which is capable of accurately sizing DNA fragments ranging from 35 to 500 bp (http://www.tcag.ca/facilities/geneticAnalysis.html. Accessed 2015 April 4). The Peak Scanner software v1.0 (Applied Biosystems, USA) was used to analyze the fragment profiles/peaks to determine the sizes of the DNA fragments, which were used to calculate SSR copy numbers. Comparison of the fragment sizes from Newfoundland isolates with those from the sequenced standards, which were included in every fragment analysis run, enabled the determination of the exact copy number of each repeat at the target SSR loci. Fragment analysis was repeated and the data was analyzed to obtain reproducible results as described previously [16]. SSR-types were assigned on the basis of the combinations of alleles for each locus and the information was used to build a dendrogram using the BioNumerics 7.1 program (Applied Maths, Inc., USA). The unweighted pair group method with arithmetic mean was used to create a minimum spanning tree using the same program, which portrays the level of divergence between strains utilizing pairwise genetic distances [23].

## Results and Discussion

The sequencing of DNA repeats using conventional methods is often challenging and is prone to artifacts, which is further exacerbated by repeats with high GC content such as those found in *Map*. In addition, currently available technologies can only determine the lengths of repeats up to ~15 bp accurately and often longer repeats cannot be measured [18]. In the case of *Map*, it has been previously reported that the analysis of 4 SSRs (L1/L2: monucleotide, and L3/L4 trinucleotide) provides enough sequence information for strain discrimination, and that one of the mononucleotide SSRs (L1) can be >14 bp in length depending on the isolate [5,14]. In our own studies we found that the sequencing of PCR products containing SSRs ~14 bp using the Sanger method was not straight forward (results not shown). Previous reports also describe similar problems where the L1 SSR had sequencing errors during analysis, leading to the misinterpretation of repeat lengths [18]. Therefore, to overcome issues associated with the analysis of *Map* SSRs, we adapted fragment analysis as a method to analyze *Map* isolates from 5 dairy farms from Newfoundland [16]. Fecal samples were collected from 18 animals, some of which showed clinical signs of Johne’s disease, displayed an immune response against *Map* in milk samples collected during previous surveys or were suspected of being infected (S1 Table). The samples were processed to obtain 18 primary fecal cultures, which were then used to establish 85 axenic cultures for use in the current study.

Before using the fragment analysis based approach, a handful of *Map* isolates (henceforth referred to as control strains) that were obtained as part of another study were subjected to SSR sequencing using the Sanger method as described previously [5,14,15,19]. Multiple sequencing runs were carried out until we could reproducibly sequence across the SSRs. This was done to determine the exact numbers of repeats at the four SSR loci for the respective isolates for subsequent use as standards for comparisons during fragment analysis. Again, we could only obtain accurate and reliable sequences for SSRs smaller than 14 bp using Sanger method (data not shown). Next, the four loci specific sets of fluorescently labeled primers were used in separate PCR reactions along with chromosomal DNA as template from the control strains described above. Since the exact numbers of SSRs present in each PCR product were known from Sanger sequencing for the control strains, they were included as size standards in all future fragment analysis experiments. The lengths of the SSRs for the K10 strain were already known (L1:19, L2:10, L3:5 and L4:5) because its genome has been sequenced [22] and could be confirmed by using fragment analysis as described below.

Chromosomal DNA was isolated from 85 axenic *Map* cultures established using samples from Newfoundland, and were used as template to obtain fluorescently labeled PCR products for fragment analysis. Comparison of each SSR PCR product with the respective standards described above provided accurate data regarding the copy number of the repeats at each locus for all 85 isolates in a short period of time (S2 Table). In the current study we were also able to analyze L2, which was not possible in a previous report that also used fragment analysis [16]. Some samples were reanalyzed to rule out ambiguities. After excluding *Map* isolates from the same animal with identical SSR profiles, a total of 68 isolates with 40 different SSR-types (M1-M44) were identified from 18 animals from 5 different Newfoundland farms (Table 1). In addition, in many cases *Map* with multiple SSR-types were isolated from the same animal (S2 Table).

**Table 1:** Details of the 40 SSR-types that were identified using fragment analysis from *Map* isolated from five Newfoundland dairy farms in the current study.

L-1 (G)^a^	L-2 (G)^a^	L-3 (GGT)^a^	L-4 (TGC)^a^	SSR-type^b^	No. of Animals with SSR-type^c^	Farm ID^d^
12	10	5	5	M1	6	A(5), C(1)
15	10	5	5	M2	5	A(4), C(1)
11	10	5	5	M3	4	A(2), C(2)
16	10	5	5	M4	4	A(3), C(1)
13	11	5	5	M5	3	A(3)
14	11	5	5	M6	3	A(2), F(1)
10	11	4	5	M7	2	C(1), E(1)
10	12	4	5	M8	2	C(2)
10	10	5	5	M9	2	A(1), C(1)
11	11	5	5	M10	2	A(1), C(1)
12	9	5	5	M11	2	A(1), F(1)
13	10	5	5	M12	2	A(1), E(1)
14	10	5	5	M13	2	A(1), E(1)
17	11	5	5	M14	2	A(1), F(1)
20	10	5	5	M15	2	A(1), E(1)
6	11	4	4	M16	1	C(1)
6	14	4	4	M17	1	D(1)
6	15	4	4	M18	1	D(1)
7	11	4	4	M19	1	C(1)
7	11	6	5	M20	1	E(1)
7	10	5	5	M21	1	A(1)
7	15	4	4	M22	1	D(1)
10	11	5	5	M23	1	A(1)
11	9	5	5	M24	1	A(1)
11	12	4	5	M25	1	C(1)
12	11	5	5	M26	1	A(1)
14	9	5	5	M27	1	A(1)
14	13	5	5	M28	1	C(1)
15	11	5	5	M29	1	A(1)
15	13	5	5	M30	1	C(1)
16	9	5	5	M31	1	A(1)
16	11	5	5	M32	1	A(1)
16	12	5	5	M33	1	A(1)
16	14	5	5	M34	1	C(1)
18	9	5	5	M35	1	F(1)
18	10	5	5	M36	1	A(1)
18	11	5	5	M37	1	F(1)
20	9	5	5	M38	1	F(1)
20	11	5	5	M39	1	A(1)
21	10	5	5	M40	1	A(1)

^**a**^The number/copies of repeats for each SSR detected in the current study are indicated.

^**b**^SSR-types were designated as M1-M40 based on the copy number of the repeats for the 4 SSR loci used in the analysis.

^**c**^The total number of animals are indicated from which *Map* with the respective SSR-types (M1-M40) were isolated.

^**d**^The assigned identity (ID) of each farm is indicated by capital letters followed by the number of animals (in parenthesis) from that farm from which *Map* with the specific SSR-type was isolated. For example, A(3) implies that 3 individual animals from Farm A had *Map* with the specific SSR-type.

**Fig 1:**
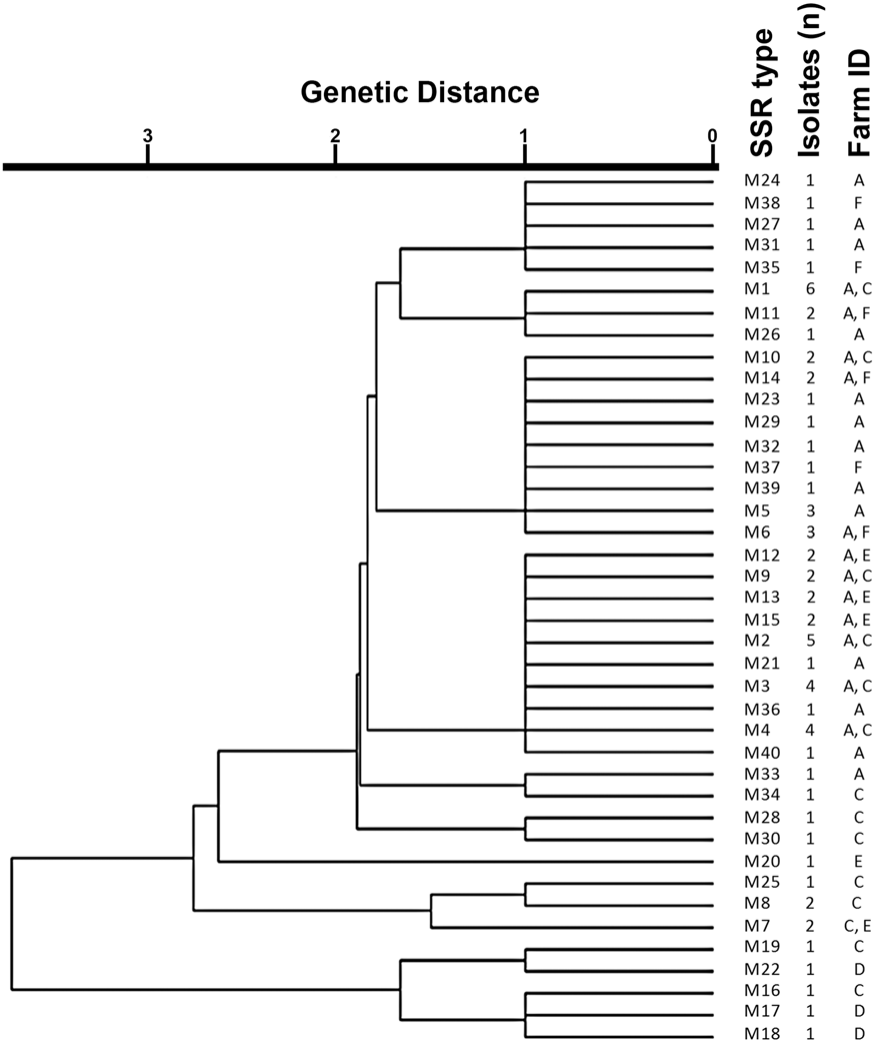
Dendrogram representing the genetic relationship between all *Map* isolates based on the 4 SSRs loci used in the analysis. The dendrogram was built using the unweighted pair group method with arithmetic mean (UPGMA) using the BioNumerics 7.1 multilocus sequence typing program. Genetic distance is indicated at the top of the dendrogram. SSR-types, number of *Map* isolates (n) and farm ID are displayed to the right side of the dendrogram.

**Fig 2:**
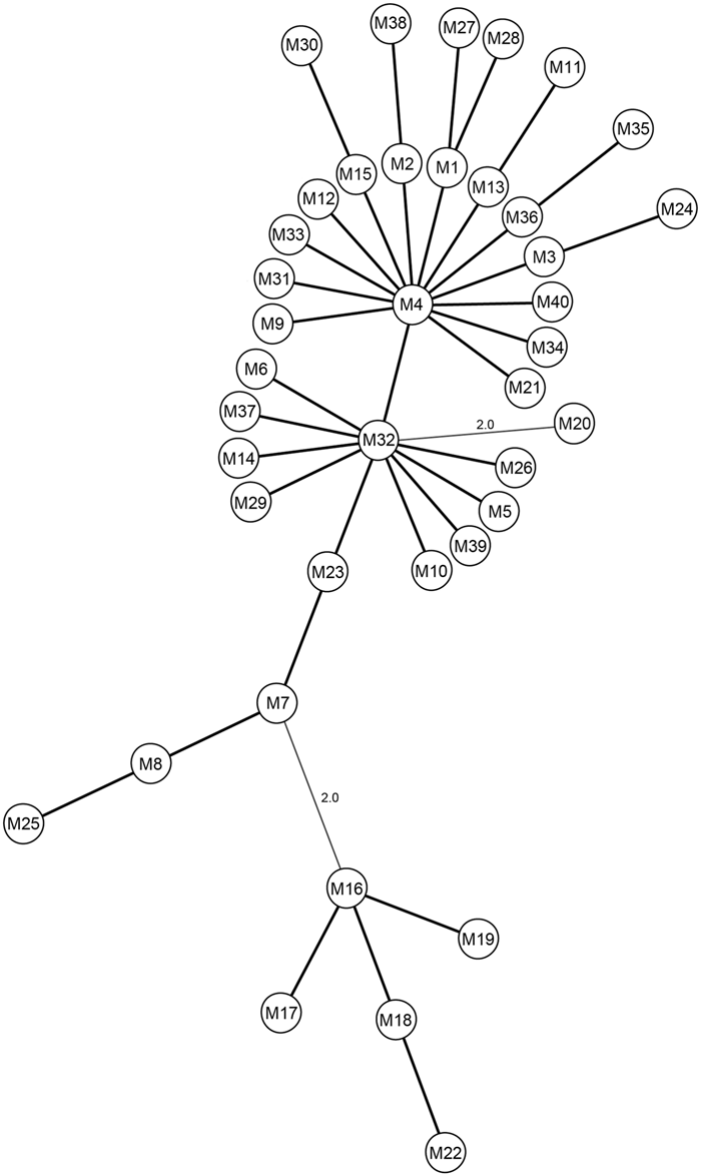
Minimum spanning tree (MST) based on the SSR profiles of the 4 loci for all 40 SSR-types identified in the current study. The tree was generated using the BioNumerics 7.1 multilocus sequence typing program and the circles represent the M1-M40 SSR-types. Thick lines represent only one variation amidst the 4 loci, whereas thin lines represent 2 differences between the 4 loci, the latter of which is indicated.

The most predominant SSR-type in the current study was M1, which was present in *Map* isolated from 6 separate animals from different farms (Table 1 and Fig 1). One reason for this observation could be the random distribution of *Map* SSR-types within farms following animal movement between farms, which is known to increase the probability of detecting similar strains on the farms involved [24]. Most SSR-types in the population were closely related to M4 and M32, differing from them in only 1–2 SSR loci (Fig 2). Overall, some farm based clustering of isolates was observed (Fig 1). A high level of diversity was seen in isolates from farm A, which alone had 25 different SSR-types out of the 40 detected. Twelve SSR-types present on farm A were also present on other farms (C, E, F) (Fig 1) suggesting probable inter herd transmission or a common source of infection. SSR-types from farm D were not detected on other farms, although they showed some level of genetic similarity with isolates from farm C (Fig 1). Animals from farm F did not exhibit any clinical signs of Johne’s disease, but tested positive for *Map* with unique SSR-types, in addition to SSR-types found on other farms also (Fig 1).

In the current study we identified 40 distinct SSR-types, some of which have not been reported previously [5,14,18,23,26]. One reason for this finding could be the insular nature of Newfoundland, which could allow for the emergence of unique SSR-types on the island. Alternatively, animals could have acquired *Map* with the respective SSR-types from other herds in Atlantic Canada, since some heifers from Newfoundland are sent to New Brunswick (NB) and Nova Scotia (NS) for rearing, and are brought back when they reach adulthood. Therefore, *Map* with these unique SSR-types could be present in Atlantic Canada, although SSR analysis has not been reported so far for a majority of herds from NB or NS for comparison. Another explanation for the distinctive genotypes could be that all SSR-types have not been reported yet as there are only a few published *Map* epidemiology studies using SSR analysis [25]. In addition, mononucleotide repeats up to 21 bp could be measured using fragment analysis, but all previous studies could only resolve SSR repeats up to 15 bp [5,14,18,23,26], which could influence their results. This critical limitation of previously used technologies could underestimate *Map* SSR diversity despite the inclusion of a large number of isolates, as it is possible that not all loci were accurately resolved [5,19,23,25]. The only other published report which used fragment analysis for typing *Map* SSRs did not include the lengths of the repeats that could be analyzed [16], but it is conceivable that resolution comparable to what was observed in the current study would have been possible.

The resolution of long SSRs by fragment analysis and the recent report showing that the technique can be multiplexed for analyzing multiple SSRs [16] further demonstrates the power and versatility of this technique for typing *Map* isolates. Future studies using techniques with better resolution capabilities and samples from other regions of North America will help to explain if the previously unidentified *Map* SSRs-types reported in the current study are unique to Newfoundland or if they were not detected due to technical limitations. In addition, results from the current study also indicated *Map* co-infection with multiple genotypes within a single animal, which has been reported as a rare event [19]. The isolation of multiple genotypes from the same animal could also be due to evolving SSR-types due to the instability associated with long DNA repeates [27]. Based on the whole genome sequences of multiple *Map* isoaltes, a recent report pointed out to the limitations of certain genotyping methods for studying *Map* strain diversity [17]. Therefore, we are also currently in the proces sequencing the genomes of a subset of our *Map* isolates for more indeapth genetic analysis (data not shown). Alternately the isolation of multiple SSR-types from the same animal might represent true co- or multiple- infections, which is important in terms of source tracking and the status of the animals involved. Therefore, studies are currently underway to address the significance and implications of the described findings.

## Supporting Information

S1 Table Details of 18 animals from Newfoundland regarding assigned identification numbers (IDs) and status of the animal from which the primary Trek-ESP II liquid cultures were derived. (PDF)

S2 Table Fragment analysis results for the 4 SSR loci of all 85 *Map* isolates derived from 18 Newfoundland animals. SSR-types were designated as M1-M40 based on their unique SSR combinations.
(PDF)
