# Supplementary material for: Typing of Mycobacterium avium Subspecies paratuberculosis Isolates from Newfoundland Using Fragment Analysis
Source: PLoS One. 2015 Apr 30;10(4):e0126071. doi: 10.1371/journal.pone.0126071 (PMC4415927; doi:10.1371/journal.pone.0126071)
Supplement: S1 Table — (PDF) [file pone.0126071.s001.pdf]

S1 Table. Details of 18 animals from Newfoundland regarding assigned identification numbers (IDs) and status of the animal from which the primary Trek-ESP II liquid cultures were derived.

Farm/animal ID[a]	Animal status/symptoms[b]
A-001	Clinical signs
A-002	Clinical signs
A-005	Clinical signs
A-006	Milk Positive for *Map*
A-007	Milk Positive for *Map*
A-008	Milk Positive for *Map*
A-009	Milk Positive for *Map*
A-010	Milk Positive for *Map*
A-011	Clinical signs
C-001	Clinical signs
C-002	Clinical signs
C-003	Clinical signs
C-004	Clinical signs
C-005	Clinical signs
D-001	Clinical signs
E-001	Clinical signs
F-001	Normal
F-002	Normal

[a] The first letter denotes the farm of origin followed by a discreet identification number assigned to each respective animal (ID: Identity).

[b] The status of each animal sampled for Trek-ESP II liquid culture for *Map* analysis is included. Animals were either asymptomatic (normal), showed clinical signs of Johne's disease or had milk samples which showed a positive immune response against *Map* during previous surveys.
