# Supplementary material for: Typing of Mycobacterium avium Subspecies paratuberculosis Isolates from Newfoundland Using Fragment Analysis
Source: PLoS One. 2015 Apr 30;10(4):e0126071. doi: 10.1371/journal.pone.0126071 (PMC4415927; doi:10.1371/journal.pone.0126071)
Supplement: S2 Table — SSR-types were designated as M1-M40 based on their unique SSR combinations. (PDF) [file pone.0126071.s002.pdf]

S2 Table. Fragment analysis results for the 4 SSR loci of all 85 MAP isolates derived from 18 Newfoundland animals. Strain types were designated as M1-M40 based on their unique SSR combinations.

Farm/animal/isolate ID[a]	DTP[b]	L-1 (G)[c]	L-2 (G)[c]	L-3 (GGT)[c]	L-4 (TGC)[c]	Strain Type
A-001-1	27	16	10	5	5	M4
A-001-2	27	10	10	5	5	M9
A-001-3	27	15	10	5	5	M2
A-001-4	27	12	10	5	5	M1
A-001-5	27	16	9	5	5	M31
A-003-1	32	13	11	5	5	M5
A-003-2	32	14	11	5	5	M6
A-003-4	32	21	10	5	5	M40
A-003-5	32	14	11	5	5	M6
A-005-1	13	12	10	5	5	M1
A-005-2	13	15	10	5	5	M2
A-005-3	13	12	10	5	5	M1
A-005-4	13	13	11	5	5	M5
A-005-5	13	11	10	5	5	M3
A-006-1	28	13	10	5	5	M12
A-006-2	28	16	12	5	5	M33
A-006-3	28	12	10	5	5	M1
A-006-4	28	12	9	5	5	M11
A-006-5	28	16	11[d]	5	5	M32
A-007-1	22	11	10	5	5	M3
A-007-2	22	12	10	5	5	M1
A-007-3	22	15	10	5	5	M2
A-007-4	22	15	10	5	5	M2
A-007-5	22	11	10	5	5	M3
A-008-1	46	18	10	5	5	M36
A-008-2	46	16	10	5	5	M4
A-008-3	46	20	10	5	5	M15
A-008-4	46	7	10	5	5	M21
A-008-5	46	17	11	5	5	M14
A-009-1	36	13	11	5	5	M5
A-009-2	36	11	11	5	5	M10
A-009-3	36	14	11	5	5	M6
A-009-4	36	20	11	5	5	M39
A-009-5	36	13	11	5	5	M5
A-010-1	49	14	10	5	5	M13
A-010-2	49	14	9	5	5	M27

A-010-3	49	12	10	5	5	M1
A-010-4	49	11	9	5	5	M24
A-010-5	49	16	10	5	5	M4
A-011-1	8	15	11	5	5	M29
A-011-2	8	12	11	5	5	M26
A-011-3	8	15	11	5	5	M29
A-011-4	8	10	11	5	5	M23
A-011-5	8	15	10	5	5	M2
C-001-1	28	10[d]	10	5	5	M9
C-001-2	28	10	10	5	5	M9
C-001-3	28	11	10	5	5	M3
C-001-4	28	10	10	5	5	M9
C-001-5	28	11	10	5	5	M3
C-002-1	28	6	11	4	4	M16
C-002-2	28	16	10	5	5	M4
C-002-3	28	15	10	5	5	M2
C-002-4	28	7	11	4	4	M19
C-002-5	28	6	11	4	4	M16
C-003-1	17	16	14	5	5	M34
C-003-2	17	14	13	5	5	M28
C-003-3	17	16	14	5	5	M34
C-003-4	17	14	13	5	5	M28
C-003-5	17	15	13	5	5	M30
C-004-1	32	10	11	4	5	M7
C-004-2	32	11	12	4	5	M25
C-004-3	32	10	11	4	5	M7
C-004-4	32	11	12	4	5	M25
C-004-5	32	10	12	4	5	M8
C-005-1	40	11	10	5	5	M3
C-005-2	40	10	12[d]	4	5	M8
C-005-3	40	12	10	5	5	M1
C-005-4	40	10	12[d]	4	5	M8
C-005-5	40	11	11[d]	5	5	M10
D-001-1	13	6	15	4	4	M18
D-001-2	13	7	15	4	4	M22
D-001-3	13	6	14	4	4	M17
E-001-1	18	10	11	4	5	M7
E-001-2	18	20[d]	10	5	5	M15
E-001-3	18	14	10	5	5	M13
E-001-4	18	13	10	5	5	M12
E-001-5	18	7	11	6	5	M20
F-001-1	19	20	9	5	5	M38
F-001-2	19	18	9	5	5	M35

F-001-3	19	12	9	5	5	M11
F-002-1	35	18	11	5	5	M37
F-002-2	35	14	11	5	5	M6
F-002-3	35	17	11	5	5	M14
F-002-4	35	17	11	5	5	M14
F-002-5	35	18	11	5	5	M37

[a] The first letter denotes the farm of origin followed by a number assigned to the animal and the last number corresponds to the single colony/isolate from 7H11 plates that were used in the analysis.

[b] DTP: Days of incubation after which growth was detected in the automated Trek-ESP II liquid culture system.

[c] Genotyping of SSRs (mononucleotide or trinucleotide) for the four loci (L): locus 1 (G repeats), locus 2 (G repeats), locus 3 (GGT repeats) and locus 4 (TGC repeats).

[d] PCR fragments from these isolates displayed two peaks (n, n+1) and exact copy number of the SSR could not be determined. For reporting and analysis purposes, the lower copy number was chosen for assigning SSR types.
